# Genomic Epidemiology of *Salmonella enterica* Serovar Hadar Strain Linked to Poultry-Associated Salmonellosis Outbreaks, United States

**DOI:** 10.3201/eid3210.260501

**Published:** 2026-10

**Authors:** Lingzi Xiaoli, Taylor Griswold, G. Sean Stapleton, Lee S. Katz, Zachary Ellison, Kaitlin A. Tagg, Hattie E. Webb, Katharine Benedict, Jessica C. Chen

**Affiliations:** Centers for Disease Control and Prevention, Atlanta, Georgia, USA (L. Xiaoli, T. Griswold, G.S. Stapleton, L.S. Katz, K.A. Tagg, H.E. Webb, K. Benedict, J.C. Chen); ASRT Inc., Smyrna, Georgia, USA (Z. Ellison)

**Keywords:** Salmonella, Salmonella enterica, Salmonella Hadar, enteric infections, bacteria, salmonellosis, molecular clock, source attribution, transmission vehicles, genomic markers, commercial poultry, backyard poultry, United States

## Abstract

Nontyphoidal *Salmonella enterica* serovar Hadar causes poultry-associated salmonellosis outbreaks in the United States. One persisting strain of *Salmonella* Hadar caused 5 multistate outbreaks resulting in ≈2,000 human cases during 2020–2023. The Centers for Disease Control and Prevention designated the strain as reoccurring, emerging, or persisting (REP), related within 26 core-genome allele differences. That REP strain has caused human illnesses by consumption of commercial poultry food products or contact with backyard poultry. To investigate the REP strain’s evolution and identify possible markers for source attribution, we performed phylogenetics and molecular clock analysis on 404 genomes subsampled from routine surveillance and outbreaks. The most recent common ancestor likely emerged in early 2018. We identified 2 clades: clade 1, associated with backyard poultry and other food sources, and clade 2, predominantly linked to commercial poultry products. We found 2 clade-specific single-nucleotide polymorphism markers; in silico screening of additional isolates supported their use for source attribution.

Nontyphoidal *Salmonella enterica* subspecies *enterica* causes >1 million human salmonellosis cases annually in the United States (*1*,*2*). Although illness is often self-limiting, with symptoms including diarrhea, fever, or abdominal pain, severe complications such as meningitis and septicemia may occur in vulnerable populations, including children <5 years of age, adults >65 years of age, immunocompromised persons, and pregnant women (*3*).

Poultry can shed *Salmonella* in their feces even when they appear healthy and clean, contaminating their living environments or food products, such as poultry and eggs (*4*,*5*). Consumption of contaminated poultry products remains the largest contributor to the overall burden of foodborne *Salmonella* infections (*6*). Similarly, contact with backyard poultry (BYP, defined as noncommercial, privately owned chickens, ducks, turkeys, or other poultry) causes more multistate outbreak–associated salmonellosis in the United States annually than any other live animal source (*7*).

Among >2,600 identified serovars of *Salmonella*, *S. enterica* serovar Hadar has emerged as a prominent contributor to multistate poultry-associated outbreaks. Its primary transmission routes include exposure to contaminated commercial poultry (CP) food products (e.g., ground turkey) and contact with BYP and their environments (*7*,*8*). Industries supplying CP and BYP are considered distinct because of differences including scale, predominant breeds produced, husbandry, and biosecurity practices, although potential overlap of upstream stages of the supply chain, such as shared hatcheries, egg suppliers, or shared feed sources, might occur (*8*–*10*). Some BYP distribution and shipping practices might contribute to further dissemination of *Salmonella* strains across species (e.g., between turkeys and chickens) when BYP hatcheries package different species together for shipping (*11*) to meet minimum customer order sizes or when feedstores display multiple species for sale in the same facility.

In 2020, a *Salmonella* Hadar strain caused outbreak A, which included >800 human infections linked to contact with BYP, and isolates were related within 15 core-genome allele differences determined by core genome multilocus sequence typing (cgMLST). During 2020–2021, *Salmonella* Hadar also caused outbreak B, linked to consumption of contaminated ground turkey in which isolates were related within 8 allele differences (*8*). Isolates from both outbreaks were closely related within 16 allele differences by cgMLST (*8*). After those outbreaks, the Centers for Disease Control and Prevention (CDC) classified isolates of *Salmonella* Hadar within a cgMLST range of 26 allele differences as a reoccurring, emerging, or persisting (REP) strain designated as REPTDK01 (*12*,*13*). REPTDK01 caused 3 more multistate outbreaks linked to contact with BYP alone (outbreaks C and D) or to BYP and CP concurrently (outbreak E). As of May 2023, ≈2,000 illnesses caused by REPTDK01 have been reported in the United States (*12*). Epidemiologic and pangenomic data suggest that REPTDK01 originated from extant populations circulating in CP and subsequently disseminated into BYP environments (*8*,*12*,*14*); however, when and where that persisting REP strain arose is unknown.

CDC developed its REP strain framework after the widespread implementation of whole-genome sequencing (WGS), which increased the ability to identify genetically related strains over a longer time period than was typically used to investigate outbreaks (*15*). The prolonged period can enable detection of related strains with broader allele differences than are typically considered for outbreak investigation. The definition of REP strains considers several factors, including information about the pathogen, the genetic relationship of investigated isolates, related epidemiologic information, and knowledge about potential sources and routes of transmission. The REP framework provides additional tools to investigate enteric illnesses and assess source tracking and overall genomic and epidemiologic trends.

To elucidate REPTDK01’s evolutionary history and identify potential genomic markers indicative of transmission vehicles, we leveraged existing epidemiologic and genomic data. Here, we conducted a molecular clock (or time tree) analysis based on single-nucleotide polymorphism (SNP) differences to estimate the emergence of the outbreak strain and investigate clade-defining SNP differences (*16*–*18*) (Appendix 1). Coupling those molecular findings with epidemiologic data, we elucidated REPTDK01’s clade association with specific transmission vehicles. We conducted our investigations consistent with applicable federal law and CDC policy: 45 C.F.R. part 46, 21 C.F.R. part 56; 42 U.S.C. §241(d); 5 U.S.C. §552a; 44 U.S.C. §3501 et seq. This work includes activities approved by the Centers for Disease Control and Prevention Institutional Review Board (approval no. 7172).

## Materials and Methods

### Data Collection

In the United States, when *Salmonella* is cultured from patient specimens, state or local public health laboratories perform WGS on the clinical isolates and upload data to PulseNet, the national molecular subtyping network for enteric disease surveillance coordinated by CDC (*19*). PulseNet uses cgMLST to assess genetic relatedness of isolates and identify potential outbreaks. For *Salmonella*, a potential outbreak cluster is flagged for investigation if >7 clinical cases (>3 cases for more rare serovars) are detected within 60 days of each other and the cases are related within 0–10 cgMLST alleles; however, that range is not used to constrain the addition of isolates (*20*). Public health officials interview patients with salmonellosis using outbreak questionnaires to obtain exposure information (e.g., foods consumed or animal contact before patient’s illness onset) to help identify transmission vehicles (*19*). During outbreak investigations, CDC or partner agencies might collect and sequence nonclinical samples from suspected foods, animals, environments at patients’ households, food production facilities, or other locations to confirm transmission vehicles. PulseNet member entities compile and analyze sequenced data from resulting nonclinical isolates. In addition, nonhuman isolates collected from routine surveillance programs such as National Antimicrobial Resistance Monitoring System (NARMS) (*21*) or ad hoc sampling are also integrated into PulseNet for analysis whenever possible.

We used a bulk dataset generated for a previous pangenomic study of REPTDK01 (*14*) as the basis for this study. That study evaluated a broader serovar Hadar dataset with carefully curated metadata, including isolates beyond REPTDK01 and from sources beyond CDC; the dataset included data generated through federal partnerships with the US Department of Agriculture and Food and Drug Administration. To study the emergence, genomics, and epidemiology of REPTDK01 more clearly, we restricted our analysis to a subsampled set of isolates from multiple sources confirmed as REPTDK01 (Appendix 2 Table 1). To analyze the overall relationships among isolates in the bulk dataset, we performed cgMLST profiling in BioNumerics version 7.6 (bioMérieux, https://www.biomerieux.com) using the EnteroBase scheme for *Salmonella* as described previously (*20*,*22*) and subsampled and analyzed REPTDK01 genomes in this study. We categorized the resulting REPTDK01 genomes into 6 groups: BYP, BYP and CP, CP, beef, pork, and unknown, using patient exposure information (e.g., individual exposure to a specific food, animal, or both) and other available metadata for food, animal, or environmental isolates. The BYP category included clinical isolates from patients who reported BYP contact as well as animal or environmental isolates obtained from samples collected from BYP or BYP habitats. The CP category included commercial poultry food isolates or clinical isolates from patients who reported consuming any type of CP food products. The BYP and CP category represented clinical isolates obtained from patients reporting exposures to both BYP and CP food products. The beef categories encompassed food isolates from cattle and pork from swine. The unknown category contained clinical or animal isolates with no available exposure information.

For REPTDK01 isolates linked to a known outbreak, we anonymized the outbreak information as A, B, C, D, and E, consistent with the nomenclature used in CDC web postings for the *Salmonella* strain (*12*). We included isolates in instances in which the incident of detection met standard criteria for opening an outbreak investigation (*19*) and the investigation determined that illnesses were likely attributed to a common source on the basis of epidemiologic information (*23*).

### Subsampling Process

We restricted our analysis to the REPTDK01 genomes within a curated collection generated for a previous pangenome study (*14*), comprising 2,021 isolates collected during 2018–2023. Most of the isolates originated during 3 consecutive years 2020–2022 (Appendix 2 Tables 1, 2). To address computational limitations and minimize oversampling of closely related isolates within the period, we stratified isolates by year of isolation and randomly subsampled ≈16% of isolates per year. In contrast, we included all available isolates for 2018, 2019, and 2023 to enhance representation. After we removed isolates not meeting our data quality criteria, the resulting subsampled dataset included 404 isolates, 78% of which were human clinical, food, animal isolates, or environmental isolates linked to 5 documented outbreaks (Appendix 1 Figure; Appendix 2 Table 2).

### Data Quality Assessment

We downloaded sequence reads from National Center for Biotechnology Information Sequence Read Archive (https:// ncbi.nlm.nih.gov/sra) (Appendix 2 Table 3). In addition to routine quality checks performed by PulseNet (*20*), we verified quality scores and read coverage using CG Pipeline version 0.5 (https://github.com/lskatz/CG-Pipeline) (*24*). We included paired-end reads in the REPTDK01 subset that had an average read coverage >30× and average quality score >28. Average quality score of 29 required 40× coverage and a score of 28 required 50× coverage. We excluded genomes that failed to meet those quality control requirements (n = 8) from subsequent analyses (Appendix 1 Figure). We generated assemblies using shovill version 1.0.9 (https://github.com/tseemann/shovill) as previously described (*14*); that study excluded contigs with coverage of <10% of the average genome coverage.

### Time Tree Analysis

To elucidate the emergence of REPTDK01 in the United States, we performed a time tree analysis. In brief, we used Lyve-SET version 1.1.4f (*25*) to construct a high-quality SNP (hqSNP)–based phylogenetic tree using default parameters for *Salmonella* and a closed chromosomal sequence of 2021K-0017 (GenBank accession no. CP093072) as the reference. We further analyzed the SNP alignment in BEAST version 2.6.3 (*26*) to generate a time tree (Appendix 1). We visualized the resulting time tree using ggtree (https://github.com/YuLab-SMU/ggtree) within R version 4.4.0 (The R Project for Statistical Computing, https://www.r-project.org).

### Identification of Clade-Defining SNPs

We performed clade-defining SNP identification by comparing a centrally related representative genome from each clade to all other representatives from that clade. First, we processed assemblies using Mashtree version 1.4.6 (*27*) to determine central genomes based on the calculated Mash distances. Using the SNP matrix generated from the previous Lyve-SET analysis, we extracted SNPs that distinguish the representative genome from clade 2 in comparison to that from clade 1. After compiling a list of candidate SNPs, we assessed their presence or absence across isolates within the same clade. We conducted χ^2^ test to statistically identify clade-specific SNPs (p<0.05). Subsequently, we focused on SNPs deemed present in almost all isolates within clade 2 and investigated their associated genes and functions through assembly annotations using Bakta version 1.9.1 (*28*).

### Pangenome Analysis

We annotated whole genome assemblies inclusive of all accessory elements using Bakta version 1.9.1 and conducted subsequent pangenomic analysis using Roary version 3.13.0 (*29*) and Scoary version 1.6.16 (*30*); we used default parameters to identify clade-defining variations in accessory genes among the REPTDK01 subset isolates.

### Genomic Marker Screening

We extracted gene sequences of *yihS* and *pdxA* from the reference genome 2021K-0017. We then used them as BLAT version 35 (*31*) references to screen 703 nonhuman REPTDK01 genomes with known epidemiologic linkages in the bulk dataset.

## Results

### Diverse Sources for *Salmonella* Hadar REPTDK01

As of May 31, 2023, the 2,021 REPTDK01 genomes from the bulk dataset were related within 26 allele differences by cgMLST (Appendix 2 Table 1). Of those, 1,544 clinical isolates (76.4%) were from patients involved in 5 multistate outbreaks (outbreaks A–E) during April 2020–May 2023; the earliest detection of the clinical isolate was in July 2019. The remaining 477 isolates were either clinical isolates not linked to a known outbreak (n = 275 [13.6%]) or isolates from other source types of food (n = 161 [8.0%]), animal (n = 35 [1.7%]), or environment (n = 6 [0.3%]).

The REPTDK01 subset included 404 isolates: 336 (83.2%) clinical, 55 (13.6%) food, and 13 (3.2%) animal isolates (Appendix 2 Table 3). Of the clinical isolates, 269 (66.6%) were associated with an outbreak. Detailed isolate metadata or exposure information was available for 163 (40.3%) of REPTDK01 subset isolates regardless of their source type (e.g., human, animal, and food), enabling categorization of the isolates into 5 exposure groups: beef (n = 1), pork (n = 1), BYP (n = 91), CP (n = 69), and BYP and CP (n = 1). We classified as unknown the remaining isolates, 173 (42.8%) outbreak-associated clinical isolates for which no exposure information was available and 68 (16.8%) not linked to outbreaks (66 clinical, 2 animal).

### Time Tree Analysis Results

Time tree analysis revealed that all but 1 REPTDK01 genomes fell within 2 well-supported clades, which shared a most recent common ancestor around early 2018 (median date February 16, 2018; 95% highest posterior density (HPD) interval April 25, 2017–July 29, 2018) (Figure). Within each clade, tree tips annotated by outbreak showed the gradual temporal evolution of REPTDK01. The full subset of 404 isolates had a median of 5 (range 0–21) allele differences characterized by cgMLST. Clade 1 consisted of 308 isolates with a median of 4 allele differences (range 0–6), and clade 2 included 95 isolates with a median of 3 allele differences (range 0–14). The corresponding hqSNP ranges were a median of 9 (range 0–40) overall, a median of 8 (range 0–33) for clade 1, and a median of 6 (range 0–24) for clade 2.

**Figure:**
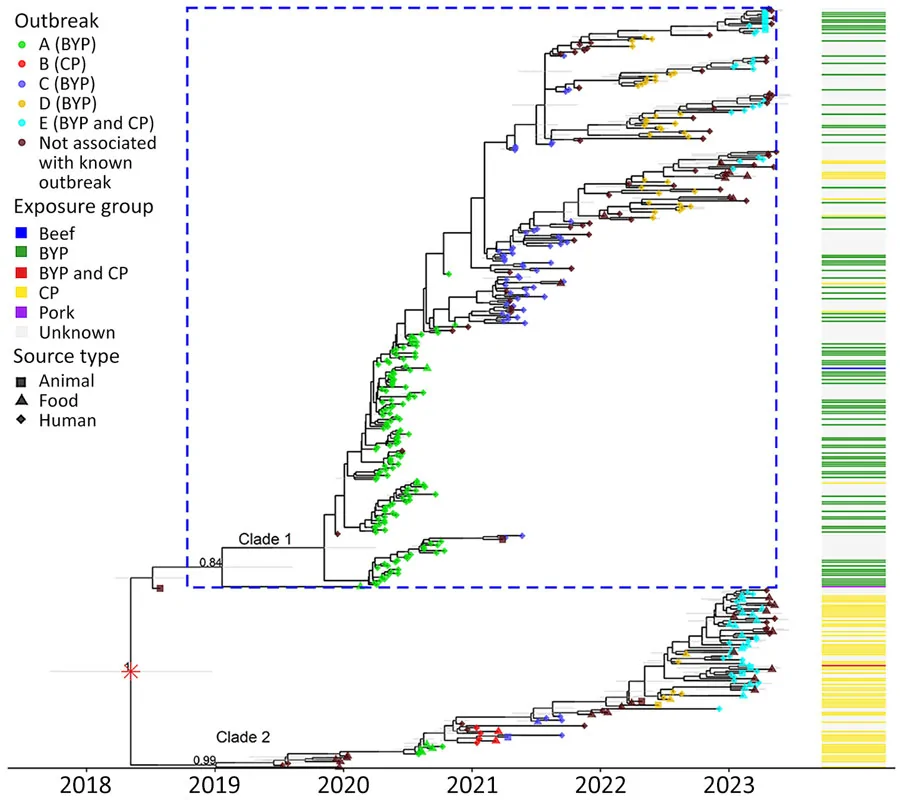
Maximum clade credibility tree of 404 persistent *Salmonella* Hadar REPTDK01 genomes from study of *Salmonella enterica* serovar Hadar strain linked to poultry-associated salmonellosis outbreaks, United States. Genomes subsampled from multistate poultry-associated outbreaks and routine surveillance revealed 2 distinct clades corresponding to separate epidemiologic linkages. The tree was generated using BEAST2 (https://www.beast2.org). Isolate tips are aligned to isolation dates and color-coded by outbreak and vehicle. Colored shape indicates source type. Red asterisk marks the most recent common ancestor, estimated to have emerged in early 2018. Posterior support values are shown for the most recent common ancestors of the 404 REPTDK01 genomes (tree root), clade 1, and clade 2. Gray horizontal bars indicate 95% highest posterior density interval for node heights. Column at right of tree indicates exposure group based on patient-related exposures and isolate metadata. Blue dashed box contains data for clade 1, comprising 308 isolates with diverse epidemiologic links to beef, pork, CP, and BYP. Isolates within clade 1 differ by a median of 8 hqSNPs (hqSNP range 0–33), and a median of 4 allele cgMLST differences (allele range 0–16). Clade 2 includes 95 isolates predominantly linked to CP; those isolates differ by a median of 6 hqSNPs (hqSNP range 0–24) or a median of 3 alleles (allele range 0–14). The full subset contains 404 isolates that differ by a median of 9 hqSNPs (hqSNP range 0–40) or 5 alleles (allele range: 0–21). BYP, backyard poultry; cgMLST, core genome multilocus sequence typing; CP, commercial poultry; hqSNP, high-quality single-nucleotide polymorphism.

The separation of 2 clades in the time tree corresponded to distinct epidemiologic linkages. Those in clade 1 exhibited greater exposure group diversity based on 104 isolates with detailed exposure information or source metadata, encompassing not only CP-associated isolates (10%, n = 11) but also those linked to BYP (88%, n = 91), beef (1%, n = 1), and pork (1%, n = 1) (Table 1). Of note, we found BYP-associated isolates only in clade 1. In contrast, isolates with detailed information in clade 2 (n = 59) were all linked to CP in some way (Table 1), including 74.5% (n = 44) from CP food, 8.5% (n = 5) from CP animals, and 17% (n = 10) from clinical isolates from case-patients exposed to CP (n = 9) or both CP and BYP (n = 1).

**Table 1:** Characteristics of isolates from genomic epidemiology study of *Salmonella* *enterica* serovar Hadar strain linked to poultry-associated salmonellosis outbreaks, United States*

Outbreak (vehicle)	Total no.	Clade 1			Clade 2	
		No. isolates	Exposure information and isolate metadata		No. isolates	Exposure information and isolate metadata
A (BYP)	140	134	54 clinical isolates with BYP exposure		6	4 CP food product isolates
			77 clinical isolates with unknown exposure			1 Clinical isolate with CP exposure
			1 beef food product isolate			1 Clinical isolate with unknown exposure
			1 pork food product isolate			
			1 CP food product isolate			
B (CP)	6	0			6	2 CP food product isolates
						1 clinical isolate with CP exposure
						3 clinical isolates with unknown exposure
C (BYP)	58	52	17 clinical isolates with BYP exposure		6	1 CP animal isolate 1 CP food product isolate
			35 clinical isolates with unknown exposure			1 clinical isolate with CP exposure
						3 clinical isolates with unknown exposure
D (BYP)	42	35	9 clinical isolates with BYP exposure		7	1 CP animal isolate 2 CP food product isolates
			26 clinical isolates with unknown exposure			2 clinical isolates with CP exposure
						2 clinical isolates with unknown exposure
E (BYP and CP)	66	24	6 BYP animal isolates		42	2 CP animal isolates 21 CP food product isolates
			5 clinical isolates with BYP exposure			4 clinical isolates with CP exposure
			1 clinical isolate with CP exposure			1 clinical isolate with CP and BYP exposure
			12 clinical isolates with unknown exposure			14 clinical isolates with unknown exposure
Not outbreak-associated	91	63	53 clinical isolates with unknown exposure		28	1 CP animal isolate 14 CP food product isolates
			1 clinical isolate with CP exposure			13 clinical isolates with unknown exposure
			8 CP food product isolates			
			1 animal isolate with unknown exposure			
Total	403	308			95	

*BYP, backyard poultry; CP, commercial poultry food products.

### *Salmonella* Hadar REPTDK01 Clades Defined by 2 SNPs

We identified 2 clade-defining SNPs using a closed genome (GenBank accession no. CP093072) from clade 1 as the reference. The first SNP led to a synonymous mutation in *pdxA*, which encodes a dehydrogenase. The second SNP, a G→A mutation in the isomerase gene *yihS*, introduced a premature stop codon (Table 1). Nearly all clade 2 isolates (97.9%) carried *pdxA* SNP, and all of them (100%) had the *yihS* SNP. Except for a truncated *yihS* gene identified in clade 2 versus wild type *yihS* in clade 1, pangenomic analysis in this study found no accessory genes distinguishing the 2 clades (data not shown).

## Discussion

Although the factors contributing to the emergence of the REPTDK01 strain of *Salmonella* Hadar remain unknown, molecular clock analysis estimated that the strain emerged in early 2018, before the earliest human case reported to PulseNet in mid-2019. Although *Salmonella* Hadar exhibits notable clonality based on previous cgMLST and pangenomic analysis (*14*), our time tree constructed on core SNPs revealed 2 clades, each harboring specific genomic variations associated with distinct epidemiologic delineations. We identified 2 clade-defining SNPs in 2 genes, *pdxA* and *yihS* (Table 2), both of which are core genes included in the cgMLST scheme used by PulseNet for outbreak detection as of July 2026 (*20*,*22*).

**Table 2:** Clade-defining SNP annotations for the *Salmonella* *enterica* serovar Hadar REPTDK01 subset used in genomic epidemiology study of *Salmonella enterica* serovar Hadar linked to poultry-associated salmonellosis outbreaks, United States†

Gene‡	Position	Ref	Alt	Type	Amino acid	Function	SNP prevalence, no. (%)		χ^2^ test
							Clade 1	Clade 2	
*pdxA*	3818146	C	T	synonymous_variant	p.Pro271Pro	Dehydrogenase	C, 308/308 (100)	C, 2/95 (2.1)	χ^2^ = 385.8, p = 6.82 × 10^–86^
								T, 93/95 (97.9)	
*yihS*	4534475	G	A	stop_gained	p.Trp290*	Isomerase	G, 308/308 (100)	A, 95/95 (100)	χ^2^ = 396.8, p = 2.78 × 10^–86^

†χ^2^ performed at 1 degree of freedom. Alt, nucleotide observed in sequence with the SNP mutation; Ref, nucleotide observed in the reference sequence; SNP, single-nucleotide polymorphism. ‡The reference genome used was 2021K-0017 (GenBank accession no. CP093072) from clade 1. χ^2^ test showed a significant difference in the distribution of the clade-defining SNP between 2 clades for each gene (p<0.05).

Although the *pdxA* mutation is synonymous, the SNP in *yihS* introduces a premature stop codon, potentially resulting in a truncated protein in clade 2 isolates. The *yihS* functions as an aldose–ketose isomerase within the conserved *yih* operon (*32*) and is implicated in sulfoquinovose metabolism, extracellular polymeric substance synthesis, and the modification of outer membrane protein antigenicity, all of which potentially influence environmental persistence under stressors such as acidity and bleach exposure (*33*–*35*). Further functional studies are needed to assess whether that *yihS* mutation contributes to clade-specific phenotypic differences in relation to differences in management across different types of poultry production systems. A 2020 study (*36*) described the expansion of a clonal group of *Salmonella* serovar Reading in turkey production. The strain was distinguished by plasmid and prophage content, as well as 2 gene disruptions, 1 in the *cirA* gene, which encodes a catecholate siderophore receptor, and 1 in a *uidA*-like gene deletion of the adjacent peptidoglycan deacetylase, *pgdA*. Those findings, combined with ours in this study, suggest mutations that inactivate genes may be a key evolutionary mechanism by which strains expand and emerge in poultry.

We observed isolates from clade 1 to have epidemiologic exposure information and isolate metadata that indicated diverse transmission vehicles including beef, pork, CP, and BYP. Of note, isolates associated with BYP exclusively belonged to clade 1, whereas isolates associated with CP predominated clade 2. That finding suggests that screening clade-defining SNPs within REPTKD01 isolates could demonstrate their linkages to a specific transmission vehicle. Preliminary in silico screening of the 2 genomic markers in an additional 703 nonsubsampled REPTDK01 isolates that had detailed epidemiologic information from the bulk dataset revealed similar patterns: all BYP-related, beef, and pork isolates along with a few CP-related and both of the CP and BYP–related isolates were assigned to clade 1, whereas most (>80%) CP-related isolates were expected to belong to clade 2 (Appendix 2 Table 4). The pangenomic study (*14*) grouped REPTDK01 isolates in 2 subgroups, JI-A and JI-C; JI-A included both BYP and CP isolates, and JI-C contained almost entirely BYP isolates. Within JI-A, the clade-defining SNPs can help further distinguish CP and BYP isolates, thus complementing those pangenomic findings. That study found that *Salmonella* Hadar subsampled for our work that carried a specific IncI1 plasmid was more often associated with BYP. Combined with the 2 genetic markers we identified, that plasmid could provide additional resolution to disentangle transmission vehicle signals of CP and BYP isolates within clade 1. Therefore, those clade-defining genomic markers could inform hypothesis generation regarding transmission vehicles when epidemiologic information is absent or limited, particularly during the early stages of outbreak investigations.

Moreover, the codetection of CP- and BYP-related isolates within clade 1 (Table 1; Figure) might indicate ongoing transmission between the 2 industries through common upstream suppliers in the production chain, rather than a single introduction from CP into BYP environments. Previous outbreak investigations have reported that BYP hatcheries occasionally source fertilized eggs and chickens used for egg production from CP industry (*10*,*37*). Some BYP hatcheries have reported sourcing specific poultry breeds (e.g., broiler or meat-producing breeds) from CP hatcheries; however, limited traceback information hinders our understanding of these upstream dynamics (*8*). Applying our proposed clade-based genomic markers for source attribution during outbreak investigations could improve future communication and collaboration with industry partners. Such mutual efforts could contribute to tailored, effective prevention measures aimed at reducing *Salmonella* transmission throughout the supply and distribution chains for poultry industries.

We noted several limitations regarding the epidemiologic information available for the full REPTDK01 dataset. First, some clinical isolates lacked detailed exposure information, which is a common issue encountered in outbreak investigations, caused by limited patient consent rates for interviews. Second, during the BYP-associated outbreak in 2020, when knowledge of the REPTDK01 strain was constrained, patient questionnaires focused solely on BYP exposure and did not necessarily include questions on exposure to CP food products for all patients, leading to inadequate attribution of epidemiologic source for those isolates. That approach has since been improved to capture additional exposure history for infected patients. Third, the REPTDK01 dataset we studied here included nonhuman isolates from retail meats and CP cecal samples collected through NARMS surveillance; it is still composed predominantly of human-associated isolates. Furthermore, NARMS surveillance sampling is focused on specific foods and food animals, so it is possible that REPTDK01 also exists in unrecognized animal populations or environments. We noted no isolates from on-farm sources or hatcheries, largely because of the difficulty in obtaining such samples. Efforts to address these limitations could include enhanced epidemiologic data collection, collaboration with experts representing poultry industries, and application of the proposed genomic markers to refine the predictive source attributions.

In conclusion, our study used a genomic epidemiology approach to better understand challenges encountered during outbreak investigations. Our findings contribute to our understanding of REPTDK01 transmission dynamics across different poultry industries and could aid in identifying transmission vehicles during outbreak investigations. Genomic markers identified in this study could suggest earlier in the investigation process whether CP or BYP industry is likely involved in prospective detections of REPTDK01 outbreaks, thereby providing earlier opportunities to coordinate across sectors and collaborate on the development of effective control and prevention measures for reducing *Salmonella* transmission.

Appendix 1 Additional methods and dataset flowchart from study of genomic epidemiology of *Salmonella enterica* serovar Hadar strain linked to poultry-associated salmonellosis outbreaks, United States.

Appendix 2 Detailed dataset descriptions and individual isolate data from study of genomic epidemiology of *Salmonella enterica* serovar Hadar strain linked to poultry-associated salmonellosis outbreaks, United States.
