# Supplementary material for: Genomic Epidemiology of Salmonella enterica Serovar Hadar Strain Linked to Poultry-Associated Salmonellosis Outbreaks, United States
Source: Emerg Infect Dis. 2026 Oct;32(10):1621–8. doi: 10.3201/eid3210.260501 (PMC13632868; doi:10.3201/eid3210.260501)
Supplement: Supplementary file 1 [file 26-0501-Techapp-s1.pdf]

Article DOI: https://doi.org/10.3201/eid3210.260501

EID cannot ensure accessibility for supplementary materials supplied by authors. Readers who have difficulty accessing supplementary content should contact the authors for assistance.

Genomic Epidemiology of *Salmonella enterica* Serovar Hadar Strain Linked to Poultry-Associated Salmonellosis Outbreaks, United States

Appendix 1

Supplemental Molecular Clock Methods

The resulting core nucleotide alignment generated in Lyve-SET v1.1.4f (*1*) was processed with Gubbins v3.0.0 (*2*) to eliminate any genome regions associated with recombination, followed by removal of invariable sites using Lyve-SET. Subsequently, the phylogenetic tree and corresponding isolation dates were analyzed with TempEST v1.5.3 (*3*) to assess temporal signal, where the best fitting root was identified and the correlation between root-to-tip divergence was 0.6463.

The resulting SNP alignment was further analyzed using BEAST v2.6.3 (*4*) to determine the most suitable tree model for this subset. We employed a strict clock framework due to the focus on a single recently evolved strain and evaluated four coalescent models: constant population, exponential population, Bayesian skyline, and exponential growth Bayesian skyline. This analysis used the filtered SNP matrix generated through Lyve-SET v1.1.4f and Gubbins v3.0.0 as previously described, with modifications made to the xml file from BEAUti v2 to include constant sites computed from the Lyve-SET alignment (https://github.com/lskatz/lyve-SET/blob/master/docs/TIPS.md#find-constant-sites) (<data id = 'filt' spec = 'FilteredAlignment' filter = '-' data = '@filtOriginal' constantSiteWeights = '102939 122691 121649 100221' />). All coalescent priors were evaluated starting with an initial clock rate estimate of 3.04×10^{-7} SNPs/site/year derived from published estimates of the *Salmonella* mutation rate, and analyses

were executed for 500,000,000 iterations with sampling every 50,000 iterations while discarding the first 10% as burn-in.

The output was evaluated using Tracer v1.7.2 (*5*), from which we selected the model producing the maximum log likelihood and producing sufficiently high effective sample size values for all parameters. TreeAnnotator in the BEAST2 suite of tools was used to generate a maximum clade credibility tree using the median height options.

References

1. Katz LS, Griswold T, Williams-Newkirk AJ, Wagner D, Petkau A, Sieffert C, et al. A comparative analysis of the Lyve-SET phylogenomics pipeline for genomic epidemiology of foodborne pathogens. Front Microbiol. 2017;8:375. PubMed https://doi.org/10.3389/fmicb.2017.00375

2. Croucher NJ, Page AJ, Connor TR, Delaney AJ, Keane JA, Bentley SD, et al. Rapid phylogenetic analysis of large samples of recombinant bacterial whole genome sequences using Gubbins. Nucleic Acids Res. 2015;43:e15. PubMed https://doi.org/10.1093/nar/gku1196

3. Rambaut A, Lam TT, Max Carvalho L, Pybus OG. Exploring the temporal structure of heterochronous sequences using TempEst (formerly Path-O-Gen). Virus Evol. 2016;2:vew007. PubMed https://doi.org/10.1093/ve/vew007

4. Bouckaert R, Vaughan TG, Barido-Sottani J, Duchêne S, Fourment M, Gavryushkina A, et al. BEAST 2.5: an advanced software platform for Bayesian evolutionary analysis. PLOS Comput Biol. 2019;15:e1006650. PubMed https://doi.org/10.1371/journal.pcbi.1006650

5. Rambaut A, Drummond AJ, Xie D, Baele G, Suchard MA. Posterior summarization in Bayesian phylogenetics using Tracer 1.7. Syst Biol. 2018;67:901–4. PubMed https://doi.org/10.1093/sysbio/syy032

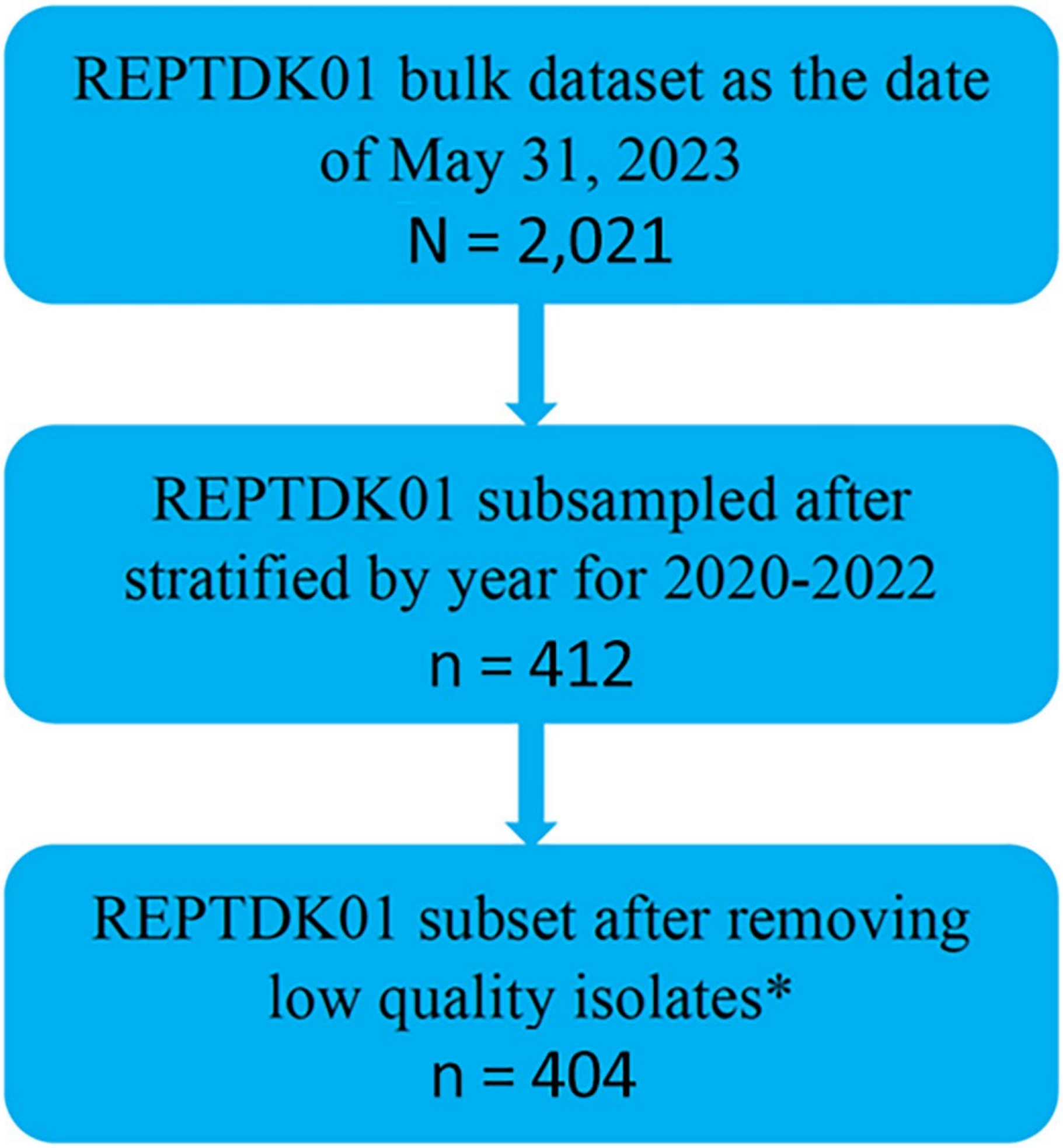

Appendix Figure. Flowchart for generating REPTDK01 subset in this study. *Eight genomes were removed due to either low quality scores (<28) or inadequate coverage to compensate for low quality scores (average quality score of 29 required ≥40x coverage and a score of 28 required ≥50x coverage).
